# Application of β-Resorcylic Acid as Potential Antimicrobial Feed Additive to Reduce *Campylobacter* Colonization in Broiler Chickens

**DOI:** 10.3389/fmicb.2017.00599

**Published:** 2017-04-06

**Authors:** Basanta R. Wagle, Abhinav Upadhyay, Komala Arsi, Sandip Shrestha, Kumar Venkitanarayanan, Annie M. Donoghue, Dan J. Donoghue

**Affiliations:** ^1^Department of Poultry Science, University of Arkansas, FayettevilleAR, USA; ^2^Department of Animal Science, University of Connecticut, StorrsCT, USA; ^3^Poultry Production and Product Safety Research Unit, United States Department of Agriculture – Agriculture Research Service, FayettevilleAR, USA

**Keywords:** *Campylobacter jejuni*, β-resorcylic acid, pre-harvest safety, chickens, colonization factors, gene expression, cell culture

## Abstract

*Campylobacter* is one of the major foodborne pathogens that result in severe gastroenteritis in humans, primarily through consumption of contaminated poultry products. Chickens are the reservoir host of *Campylobacter*, where the pathogen colonizes the ceca, thereby leading to contamination of carcass during slaughter. A reduction in cecal colonization by *Campylobacter* would directly translate into reduced product contamination and risk of human infections. With increasing consumer demand for antibiotic free chickens, significant research is being conducted to discover natural, safe and economical antimicrobials that can effectively control *Campylobacter* colonization in birds. This study investigated the efficacy of in-feed supplementation of a phytophenolic compound, β-resorcylic acid (BR) for reducing *Campylobacter* colonization in broiler chickens. In two separate, replicate trials, day-old-chicks (Cobb500; *n* = 10 birds/treatment) were fed with BR (0, 0.25, 0.5, or 1%) in feed for a period of 14 days (*n* = 40/trial). Birds were challenged with a four-strain mixture of *Campylobacter jejuni* (∼10^6^ CFU/ml; 250 μl/bird) on day 7 and cecal samples were collected on day 14 for enumerating surviving *Campylobacter* in cecal contents. In addition, the effect of BR on the critical colonization factors of *Campylobacter* (motility, epithelial cell attachment) was studied using phenotypic assay, cell culture, and real-time quantitative PCR. Supplementation of BR in poultry feed for 14 days at 0.5 and 1% reduced *Campylobacter* populations in cecal contents by ∼2.5 and 1.7 Log CFU/g, respectively (*P* < 0.05). No significant differences in feed intake and body weight gain were observed between control and treatment birds fed the compound (*P* > 0.05). Follow up mechanistic analysis revealed that sub-inhibitory concentration of BR significantly reduced *Campylobacter* motility, attachment to and invasion of Caco-2 cells. In addition, the expression of *C. jejuni* genes coding for motility (*motA, motB, fliA*) and attachment (*jlpA, ciaB*) was down-regulated as compared to controls (*P* < 0.05). These results suggest that BR could potentially be used as a feed additive to reduce *Campylobacter* colonization in broilers.

## Introduction

*Campylobacter* contamination of food products is the leading cause of bacterial foodborne illness worldwide (Crim et al., 2015; Mangen et al., 2016). *Campylobacter*, in particular, *Campylobacter jejuni*, is the second most commonly reported foodborne pathogen in the USA with an annual incidence of 13.45 per 100,000 resulting in approximately 1.3 million infections annually (Crim et al., 2015). Actual cases are probably higher than these estimates due to under-reporting or sick individuals not seeking medical attention (Mead et al., 1999; Samuel et al., 2004). *Campylobacter* causes mild to severe gastroenteritis, fever, vomiting, and diarrhea in patients. In a minority of cases, it leads to more serious Guillain–Barré syndrome, reactive arthritis, or irritable bowel syndrome (Spiller, 2007; Gradel et al., 2009). Epidemiological studies have shown that the major risk factors associated with *Campylobacter* infections are improper handling and consumption of chicken or other food products cross-contaminated with poultry meat or juice during food preparation (Rosenquist et al., 2003; Friedman et al., 2004; Danis et al., 2009). The low infectious dose of *C. jejuni* (∼500 CFU) further raises public health concerns since only a few microorganisms are needed to cause the infection (Black et al., 1988).

*Campylobacter* sp. colonizes the gastrointestinal tract of chickens by the third to fourth week of age as a commensal organism (Annan-Prah and Janc, 1988; Stern et al., 1988; Humphery et al., 1993; Dhillon et al., 2006). Various studies have reported enteric colonization up to 10^8^ CFU/g of cecal contents in birds (Beery et al., 1988; Achen et al., 1998). Product contamination mostly occurs during slaughtering of chickens (Berrang et al., 2000; Herman et al., 2003; Reich et al., 2008; Boysen et al., 2016). Therefore, effective strategies to control *Campylobacter* in poultry flocks at the farm level are needed to reduce product contamination and the incidence of campylobacteriosis in humans (Rosenquist et al., 2003; Arsenault et al., 2007; Reich et al., 2008).

A variety of pre-harvest strategies have been employed to reduce *Campylobacter* in poultry with varied degree of success. These include feeding birds with bacteriophages (Carrillo et al., 2005; Wagenaar et al., 2005), bacteriocins (Stern et al., 2005; Svetoch and Stern, 2010), probiotics (Santini et al., 2010; Arsi et al., 2015; Shrestha et al., 2017), and vaccination (Buckley et al., 2010; Chintoan-Uta et al., 2016). With increasing consumer demand for safe and natural products with minimal preservatives, significant research is being conducted to explore the potential of natural antimicrobials for controlling *C. jejuni* in chickens (Hermans et al., 2011b).

Since ancient times, plant compounds have been used for improving shelf life and microbiological safety of food. The antimicrobial activity of several phytochemicals has been previously reported (Burt, 2004; Holley and Patel, 2005; Upadhyay et al., 2014). β-resorcylic acid (BR; 2, 4 dihydroxybenzoic acid) is a phytophenolic compound that is widely distributed among the angiosperms as a secondary metabolite to protect plants against pathogens (Friedman et al., 2003). It is classified under “Everything Added to Food in the United States” (EAFUS; Cas no. 89-86-1) by the US-FDA (U. S. FDA EAF 3045; U. S. Food and Drug Administration, 2013). Previous research has shown that BR is effective in reducing major foodborne pathogens, including *Salmonella* (Mattson et al., 2011), *Listeria monocytogenes* (Upadhyay et al., 2013), and *Escherichia coli* O157:H7 (Baskaran et al., 2013) in food products. However, its efficacy in reducing *C. jejuni* in chickens has not been determined.

The objective of this study was to investigate the efficacy of in-feed supplementation of BR in reducing *C. jejuni* colonization in broiler chickens. In addition, the effect of BR on the various virulence factors critical for *Campylobacter* colonization in chickens was investigated.

## Materials and Methods

### *Campylobacter* Strains and Culture Conditions

Four wild strains (S-1, S-3, S-4, S-8) of *C. jejuni* originally isolated from the commercial broilers were used in the study. Each strain was grown separately in 10 ml of *Campylobacter* enrichment broth (CEB, International Diagnostics Group, Bury, Lancashire, UK) for 48 h at 42°C under microaerophilic condition (5% O_2_, 10% CO_2_, and 85% N_2_).

### Anti-*Campylobacter* Efficacy of BR in Chicken Cecal Contents

The antibacterial activity of BR against *C. jejuni* in cecal contents was investigated as described before (Kollanoor Johny et al., 2010). Cecal contents from broiler chickens were collected and autoclaved at 121°C for 15 min. The autoclaved cecal contents (10 ml) were inoculated with *C. jejuni* to ∼10^8^ CFU/ml. Different concentrations of treatment solution were prepared by dissolving appropriate quantities of BR (Sigma-Aldrich, Co., St. Louis, MO, USA) in CEB to make a final concentration of 0.25, 0.50, and 1% BR. *Campylobacter* enrichment broth was used as a control. Then, 100 μl of cecal content inoculated with the four-strain mixture of *C. jejuni* and 900 μl of respective treatment solutions were added in tubes and incubated at 42°C under microaerophilic condition for 24 h. Duplicate samples were serially diluted (1:10) in Butterfield’s phosphate diluent (BPD, 0.625 mM potassium dihydrogen phosphate, pH 7.2) and plated at 0, 8, 24 h on *Campylobacter* line agar (CLA; Line, 2001), followed by incubation at 42°C for 48 h to enumerate the surviving *C. jejuni.*

### Bird Housing, *Campylobacter* Challenge, and Enumeration

Two *in vivo* studies were conducted with a total of 80 birds. In each trial, 40 day of hatch broiler chicks (Cobb500) were obtained from a commercial hatchery. All the experiments were approved by the Institutional Animal Care and Use Committee of the University of Arkansas and recommended guidelines were followed for animal handling. Birds were weighed and randomly allocated to one of four treatments groups (0, 0.25, 0.5, 1% BR) (*n* = 10 chicks/treatment/trial). BR was thoroughly mixed in mash feed using a commercial feed mixer and was fed throughout the 14-day study period along with *ad libitum* water. The feed consumption, initial and final body weights of individual birds was recorded during the study. Birds were challenged via oral gavage with a cocktail of four wild strains of *C. jejuni* (6 Log CFU/ml; 250 μl/bird) on day 7. On day 14, cecal contents were collected aseptically, serially diluted and plated on CLA for enumeration of *Campylobacter* (Cole et al., 2006). Confirmation of *Campylobacter* colonies was made with a latex agglutination test (Scimedx, Co., Dover, NJ, USA).

### Determination of SIC of BR

The sub-inhibitory concentration (SIC) of BR was determined as described previously (Upadhyay et al., 2012). Briefly, two-fold dilutions of BR (0, 0.1, 0.05, 0.025, 0.0125, 0.00625, and 0.003125%) were made in a sterile 24-well polystyrene plate (Costar, Corning, NY, USA) containing CEB followed by inoculation with wild type S-8 strain of *C. jejuni* (∼10^6^ CFU/ml). The plates were incubated at 42°C under microaerophilic condition for 24 h and the growth of *C. jejuni* was enumerated. The highest concentration of BR that did not inhibit the growth of *C. jejuni* as compared to controls was selected as the SIC of the compound.

### Motility Assay

The effect of BR on the motility of *C. jejuni* was determined as described previously (Upadhyay et al., 2012). Motility test agar plates (Becton, Dickinson and Company, Sparks, MD, USA) with or without (control) SIC of BR were prepared and stab inoculated in the center with 5 μl culture of *C. jejuni* strain S-8 (6 log CFU). The plates were incubated for 48 h at 42°C under microaerophilic condition and the zone of motility were measured.

### Adhesion and Invasion Assays

The effect of SIC of BR on *C. jejuni* adhesion to and invasion of human Caco-2 cells was investigated as described previously (Moroni et al., 2006) with modifications. Human enterocytes were maintained in DMEM (VWR Life Science, Rochester, NY, USA) containing 10% fetal bovine serum (VWR Life Science, Rochester, NY, USA). Caco-2 cells were grown in sterile 24-wells culture plates (∼10^5^ cells per well) to form monolayer at 37°C in a humidified, 5% CO_2_ incubator. The Caco-2 monolayer was inoculated with the mid-log phase of *C. jejuni* S-8 (∼6 Log CFU/ml; multiplicity of infection 10:1) either in the presence or absence of SIC of BR. For the adhesion assay, infected monolayer (after an hour of incubation at 42°C under microaerophilic condition) was rinsed twice in minimal media and lysed with 0.1% Triton-X 100 (Sigma-Aldrich, Co., St. Louis, MO, USA) for 15 min. The enumeration of adherent *C. jejuni* S-8 was made by serial dilution and plating on CLA. For the invasion assay, infected cells (after an hour of incubation) were rinsed twice in minimal media followed by 2 h of incubation in whole media containing gentamicin (200 μg/ml) (Sigma-Aldrich, Co., St. Louis, MO, USA) to kill extracellular bacteria. After incubation, the cells were processed for enumerating the number of invaded bacteria as described above.

### RNA Isolation, cDNA Synthesis, and Real-Time Quantitative PCR

The effect of SIC of BR on the expression of *Campylobacter* chicken colonization genes was studied using real-time quantitative PCR (RT-qPCR) as described previously (Woodall et al., 2005; Upadhyay et al., 2012). *C. jejuni* strain S-8 was cultured with or without SIC of BR in CEB at 42° C under microaerophilic condition and total RNA was extracted at mid-log phase (10 h) using RNA mini kit (Invitrogen, Carlsbad, CA, USA). The complementary DNA (cDNA) was made using iScript cDNA synthesis kit (Bio-Rad) after DNase treatment (Thermo Fisher Scientific, Carlsbad, CA, USA). All the primers in our study (**Table 1**) were designed from published Gene Bank *C. jejuni* sequences using Primer 3 software (National Center for Biotechnology Information) and obtained from Integrated DNA Technologies. The cDNA was used as the template and the amplified product was detected by SYBR Green reagent (iQ SYBR Green Supermix, Bio-Rad). The primer specificity was tested using NCBI-Primer BLAST, melt curve analysis and *in silico* PCR (Bikandi et al., 2004). Data were normalized to endogenous control (16S rRNA) and comparative analyses of expression of candidate genes were determined using the comparative critical threshold (ΔΔCt) method on Quant Studio 3 real-time PCR system (Applied Biosystems, Thermo Fisher Scientific, Carlsbad, CA, USA).

**Table 1:** Primers used for gene expression analysis using real-time quantitative PCR.

Gene with accession no.	Primer	Sequence (5′–3′)
16S-rRNA (NC_002163.1)	Forward	5′-ATAAGCACCGGCTAACTCCG-3′
(product length 78 bp)	Reverse	5′-TTACGCCCAGTGATTCCGAG-3′
*luxS* (NC_002163.1)	Forward	5′-AGTGTTGCAAAAGCTTGGGA-3′
(product length 106 bp)	Reverse	5′-GCATTGCACAAGTTCCGCAT-3′
*motA* (NC_002163.1)	Forward	5′-AGCGGGTATTTCAGGTGCTT-3′
(product length 75 bp)	Reverse	5′-CCCCAAGGAGCAAAAAGTGC-3′
*motB* (NC_002163.1)	Forward	5′-AATGCCCAGAATGTCCAGCA-3′
(product length 51 bp)	Reverse	5′-AGTCTGCATAAGGCACAGCC-3′
*fliA* (NC_002163.1)	Forward	5′-AGCTTTCACGCCGTTACGAT-3′
(product length 56 bp)	Reverse	5′-TCTTGCAAAACCCCAGAAGT-3′
*ciaB* (NC_002163.1)	Forward	5′-TCTCAGCTCAAGTCGTTCCA-3′
(product length 50 bp)	Reverse	5′-GCCCGCCTTAGAACTTACAA-3′
*jlpA* (NC_002163.1)	Forward	5′-AGCACACAGGGAATCGACAG-3′
(product length 66 bp)	Reverse	5′-TAACGCTTCTGTGGCGTCTT-3′
*cadF* (NC_002163.1)	Forward	5′-CGCGGGTGTAAAATTCCGTC-3′
(product length 135 bp)	Reverse	5′-TCCTTTTTGCCACCAAAACCA-3′

### Statistical Analyses

The *Campylobacter* mean CFUs were logarithmically transformed (Log CFU) to maintain the homogeneity of variance (Byrd et al., 2001). For all the *in vitro* experiments, duplicate samples were used and the assay was repeated three times. The data from trial 1 and trial 2 (*in vivo* study) and all *in vitro* experiments were pooled and analyzed using PROC MIXED procedure in SAS software (version 9.4, SAS Institute, Inc., Cary, NC, USA). The treatment means were separated by least square means, and a probability of *P* < 0.05 was required for statistical significance.

## Results

### Anti-*Campylobacter* Efficacy of BR in Chicken Cecal Contents *In vitro*

**FIGURE 1:**
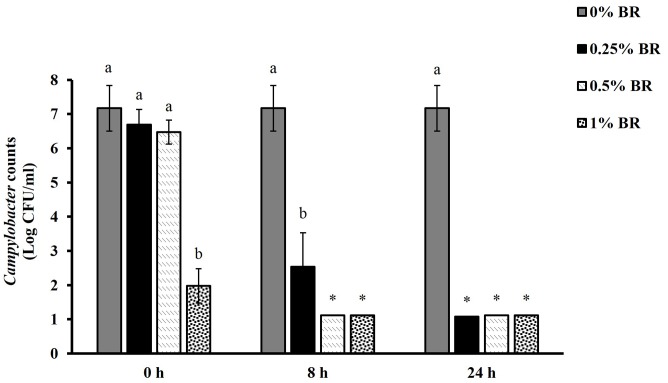
**Reduction of *Campylobacter* counts in cecal contents by different concentrations (0, 0.25, 0.50, and 1%) of BR at 0, 8, and 24 h**. Results are averages of three independent experiments, each containing duplicate samples (mean and SEM). Bars with different letters represent a significant difference between treatments (*P* < 0.05). ^∗^Indicates *Campylobacter* counts below the detection limit (1 Log CFU/ml).

**Figure 1** shows the effect of BR in reducing *Campylobacter* in chicken cecal contents *in vitro*. Among the various BR treatments, only 1% BR treatment significantly reduced *Campylobacter* counts at 0 h by 5.2 Log CFU/ml. At 8 h all the BR treatments significantly reduced *Campylobacter* populations in chicken cecal contents. Both 0.5 and 1% BR treatments reduced the counts below detection limit (1 Log CFU/ml) whereas 0.25% BR significantly reduced the counts by 4.6 Log CFU/ml compared to control. All the doses of BR reduced counts below detection limit after 24 h.

### Effect of BR on *C. jejuni* Cecal Colonization and Average Body Weight Gain in Broiler Chickens

**FIGURE 2:**
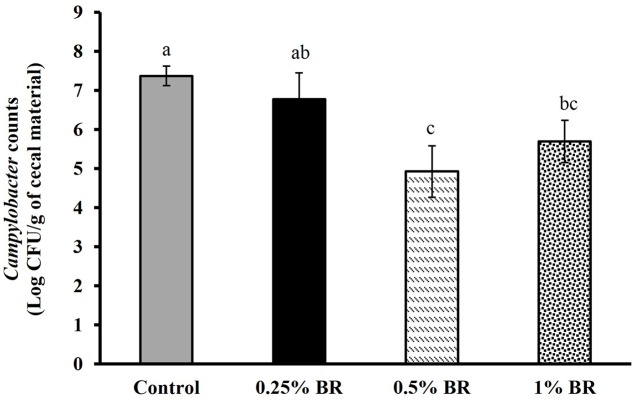
**Effect of BR on cecal *Campylobacter* counts in 14 days old broiler chickens**. Results are averages of two independent experiments, each containing 10 birds/treatments (mean and SEM). Bars with different letters represent a significant difference between treatments (*P* < 0.05).

**FIGURE 3:**
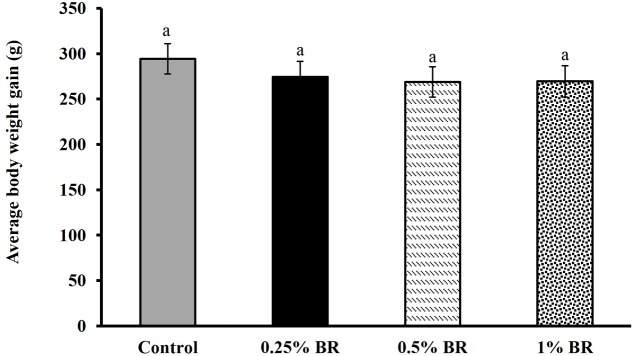
**Effect of BR on body weight gain in broiler chickens**. Results are averages of two independent experiments, each containing 10 birds/treatments (mean and SEM). Bars with different letters represent a significant difference between treatments (*P* < 0.05).

The effect of BR supplementation on *Campylobacter* cecal colonization in broilers is presented in **Figure 2**. In case of control, an average *Campylobacter* colonization of ∼7.5 Log CFU/g of cecal contents was observed on day 14. Supplementation of BR in feed at 0.5 and 1% level reduced cecal *C. jejuni* counts by ∼2.5 and 1.7 Log CFU/g, respectively as compared to the controls. However, BR supplementation at 0.25% did not significantly reduce *C. jejuni* counts in chickens. There was no significant difference in body weight gains in birds fed with BR compared to control birds at the end of 14 days (**Figure 3**).

### Estimation of Sub-inhibitory Concentration (SIC) of BR for Mechanistic Analysis

Based on growth curve analysis, the concentration of BR that did not significantly inhibit the growth of *C. jejuni* was 0.0125% and was selected as the SIC for subsequent mechanistic analysis (data not shown). Since DMSO was used as the diluent for BR, its effect on various colonization factors of *C. jejuni* was also studied.

### Effect of SIC of BR on *C. jejuni* Colonization Factors (Motility, Attachment, and Invasion of Caco-2 Cells)

**FIGURE 4:**
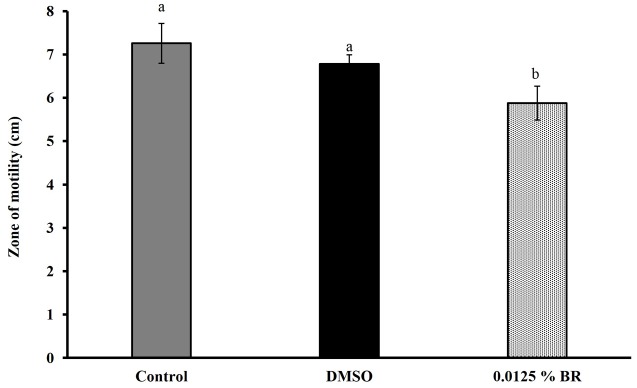
**Effect of SIC of BR on the motility of *Campylobacter jejuni***. Results are averages of three independent experiments, each containing duplicate samples (mean and SEM). Bars with different letters represent a significant difference between treatments (*P* < 0.05).

**FIGURE 5:**
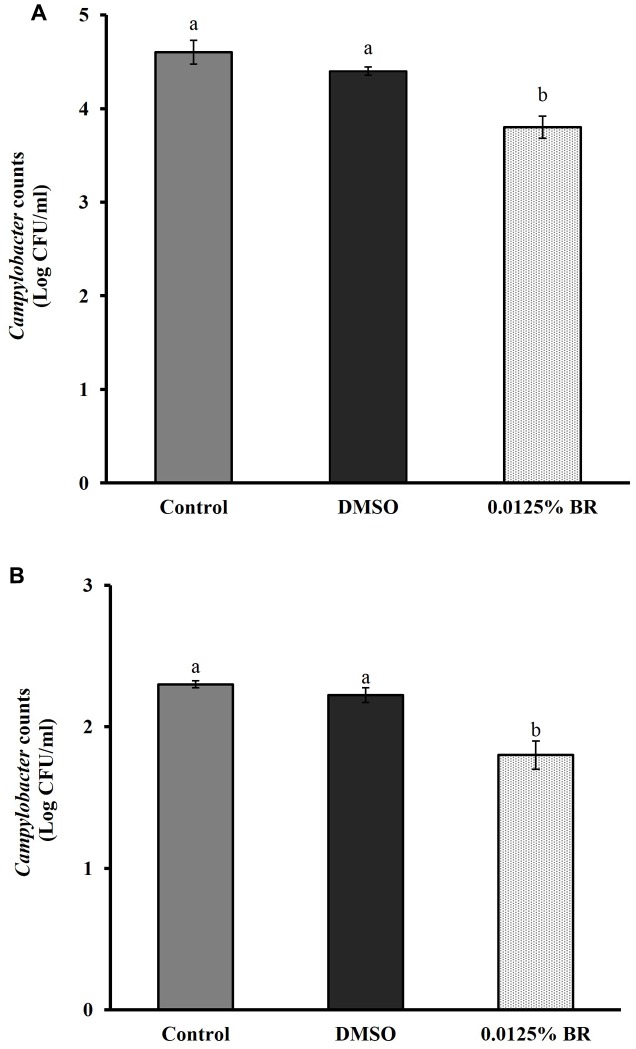
**Effect of BR on *C. jejuni*** **(A)** adhesion to and **(B)** invasion of human enterocytes. Results are averages of three independent experiments, each containing duplicate samples (mean and SEM). Bars with different letters represent a significant difference between treatments (*P* < 0.05).

The addition of BR did not change the pH of motility medium or cell culture medium (*P* > 0.05). **Figure 4** shows the effect of 0.0125% BR on *C. jejuni* motility. The SIC of BR reduced the zone of motility of *C. jejuni* to ∼5.9 cm (20% reduction) as compared to control that had a zone of ∼7.3 cm. In addition, BR also reduced *C. jejuni* attachment to and invasion of Caco-2 cells (**Figures 5A,B**). The adhesion of *C. jejuni* to Caco-2 cells was reduced by ∼0.7 Log CFU/ml (16%) as compared to control (**Figure 5A**). Similarly, *C. jejuni* invasion of Caco-2 cells was reduced by ∼0.5 Log CFU/ml (35%) as compared to control (**Figure 5B**). The DMSO treatment did not significantly affect the motility (**Figure 4**), adhesion (**Figure 5A**), or invasion of *C. jejuni* (**Figure 5B**). Taken together, these results show that BR exerts inhibitory effect on major colonization factors of *C. jejuni.*

### Effect of BR on Expression of *C. jejuni* Poultry Colonization Genes

**FIGURE 6:**
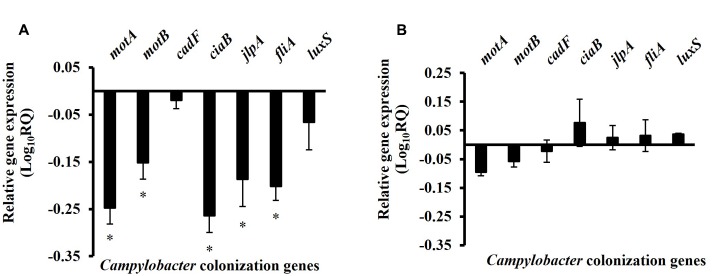
**The effect of** **(A)** 0.0125% BR and **(B)** DMSO on the expression of chicken colonization genes of *C. jejuni*. 16S-rRNA served as endogenous control. Results are averages of three independent experiments, each containing duplicate samples (mean and SEM). ^∗^Indicates significantly down-regulated genes (*P* < 0.05).

The effect of SIC of BR on the expression of *Campylobacter* colonization genes is shown in **Figure 6A**. RT-qPCR revealed that BR reduced the transcription of genes critical for motility (*motA*, *motB, fliA*), adhesion and invasion (*jlpA* and *ciaB*) of *Campylobacter* as compared to control. However, other genes coding for quorum sensing (*luxS*) and attachment (*cadF*) were not significantly modulated. The DMSO treatment did not affect the expression of the tested genes (*P* > 0.05) (**Figure 6B**).

## Discussion

Despite substantial efforts, *C. jejun*i remains a leading biological contaminant of chicken products (Hermans et al., 2011b). Since *Campylobacter* resides primarily in the cecal crypts of birds, effective pre-harvest control strategy that reduces pathogen colonization in the cecal environment could potentially reduce the risk of fecal shedding and subsequent product contamination (Beery et al., 1988). An antimicrobial treatment that can be administered through feed represents a practical method for controlling pathogen colonization in birds. With increasing consumer demand for antibiotic free chickens, plant derived compounds or phytochemicals represent a large untapped resource that can serve as a safe and effective antibiotic alternative for controlling pathogens in birds.

Since degradation of chemicals in cecal contents is a potential concern, we investigated the anti-*Campylobacter* efficacy of BR in presence of cecal contents as a first step before conducting *in vivo* studies in broilers. We observed that BR is effective at reducing or eliminating *C. jejuni* in presence of cecal contents. The BR treatments reduced *C. jejuni* in a dose-dependent manner with highest tested dose (1%) showing significant reductions immediately (0 h), while the lower doses (0.25 and 0.5%) were effective after 8 h (**Figure 1**). Therefore, we selected these concentrations for testing their anti-*Campylobacter* efficacy in broilers.

Previous research has shown that a 2-Log reduction of *Campylobacter* counts from the poultry would produce up to 95% reduction in the risk of campylobacteriosis in humans (Nauta et al., 2016). In our study, *C. jejuni* colonized the birds effectively (∼7.5 Log CFU/g cecal contents in controls) and the compound consistently reduced *C. jeuni* colonization in both trials, therefore each data were combined (**Figure 2**). Supplementation of BR at 0.5 or 1.0% in feed significantly reduced enteric *Campylobacter* counts by at least 1.5 Log CFU/g when compared with positive control, indicating that BR could potentially reduce risk of subsequent human infections by reducing *Campylobacter* colonization in broilers. Previously, Upadhyaya et al. (2014) reported that supplementation of BR at 1% in feed reduced *Salmonella enteritidis* colonization in cecum, liver and crop by at least 1.5 Log CFU/g in 21 day broiler chickens suggesting that BR has a broad antimicrobial activity that includes major poultry associated foodborne pathogens. Structure-activity studies suggest that the antimicrobial activity of BR is associated with presence of carboxyl and hydroxyl groups on the phenol ring in its structure (Friedman et al., 2003).

Although found to be effective in controlling foodborne pathogen, selection of BR dosage is critical for optimal antimicrobial efficacy in poultry. We observed that feeding BR at level of 1.5% led to slower weight gain in birds (data not shown). Józefiak et al. (2010) reported that supplementation of benzoic acid (dehydroxylated form of BR) at 0.2% depressed the growth of broiler chickens. However, in another study, supplementation of benzoic acid at 0.1% in feed improved the performance in turkey poults (Giannenas et al., 2014). These researchers also observed an increase in lactic acid bacteria and decrease in coliform bacteria in the ceca. There are only a few studies on the absorption, metabolism, and effect of BR on poultry gut. Beta-resorcylic acid has a moderate dissociation constant similar to benzoic acid, and as a weak acid remains in a non-dissociated form in the stomach and intestine (Mattoo, 1959; Milne, 1965). Therefore, it might be acting through similar mechanism(s). It is possible that feeding higher concentration of BR could modulate poultry gut environment leading to reduced appetite and suppressed growth in chickens.

To investigate the potential mechanism of action of BR on *Campylobacter*, we evaluated the effect of BR at its SIC, on various virulence attributes of *C. jejuni. C. jejuni* strain S-8 was randomly selected from the four-strains for mechanistic analysis. Since SICs are not bacteriostatic or bactericidal, the results we observed in our phenotypic assays are not due to killing of *Campylobacter* but potentially due to modulation of its pathophysiology. Motility of *Campylobacter* is one of the essential factors for colonization in poultry gut (Morooka et al., 1985; Hermans et al., 2011a). We found that presence of BR in the medium reduced *Campylobacter* motility as compared to controls. Similar results of reduced *Campylobacter* motility were observed when *Campylobacter* was exposed to citrus extract (Castillo et al., 2014) and berries (Salaheen et al., 2014). Since a chicken cecal epithelial cell line is not commercially available, we used the well-established human epithelial cells (Caco-2) for conducting attachment and invasion assays. Previously, Byrne et al. (2007) showed that *C. jejuni* attaches and invades both human epithelial cells and primary chicken enterocytes with similar efficiency. Our results from the cell culture revealed that BR reduced *C. jejuni* adhesion and invasion of human enterocytes by 0.7 and 0.5 Log CFU/ml respectively compared to control. This reduction was similar to that observed with other phytochemicals such as berry extracts (Salaheen et al., 2014), and extracts from *Acacia farnesiana*, *Artemisia ludoviciana*, *Opuntia ficus-indica*, and *Cynara scolymus* (Castillo et al., 2014). Since *Campylobacter* adhesion to epithelial cells is an important step for colonization (Jin et al., 2001; Hermans et al., 2011a), a reduction in this virulence attribute could potentially reduce colonization in birds. Bezek et al. (2016) had similar findings with extracts from *Euodia ruticarpa*. In addition, these researchers observed that other virulence attributes, including biofilm formation and quorum sensing were also affected by the phytochemical.

It is previously reported that the SIC of antimicrobials modulates the expression of various virulence proteins and associated genes in bacteria thereby resulting in changes in their pathophysiology and virulence (Goh et al., 2002; Tsui et al., 2004; Qiu et al., 2010; Upadhyay et al., 2012, 2014; Maisuria et al., 2016). To study if similar mechanisms exist in *Campylobacter*, we investigated the effect of BR on various genes of *Campylobacter* that are known to facilitate colonization in poultry. *motA*, *motB*, and *fliA* genes are critical for *Campylobacter* motility (Nachamkin et al., 1993; Wassenaar et al., 1993; Fernando et al., 2007). *motA* and *motB* code for flagella motor protein, while *fliA* codes for flagella biosynthesis protein. Similarly, *cadF* along with the genes *jlpA* and *ciaB* facilitate *C. jejuni* adherence onto the intestinal cells (Hermans et al., 2011a). The transcription level of genes coding for motility (*motA, motB, fliA*) and attachment (*jlpA*, *ciaB*) was downregulated as revealed in RT-qPCR results, thus indicating that the anti-*Campylobacter* colonization effect observed with BR could be mediated via downregulation of critical colonization genes in the pathogen.

## Conclusion

β-resorcylic acid supplementation in feed reduced *Campylobacter* colonization in birds without affecting their body weight gain or feed conversion ratio. Mechanistic analysis using standard bioassays, cell culture and gene expression analysis showed that the reduction in *Campylobacter* colonization in birds could at least partially be attributed to modulation of critical colonization factors, virulence proteins and associated genes in the pathogen. Thus, BR could potentially be used as a feed supplement to control *C. jejuni* colonization in broiler chickens. Although, the results from this study are encouraging, follow-up studies investigating the efficacy of BR in market age birds and cost-benefit analysis of feed application are warranted.

## Disclaimer

Mention of a trade name, proprietary product, or specific equipment does not constitute a guarantee or warranty by the USDA and does not imply its approval to the exclusion of other products that may be suitable.

## Author Contributions

BW and DD designed the study. BW, AU, KA, SS conducted the experiments. BW and AU wrote the manuscript. KV, AD, and DD critically analyzed and revised the manuscript.

## Conflict of Interest Statement

The authors declare that the research was conducted in the absence of any commercial or financial relationships that could be construed as a potential conflict of interest.
